# IL-22, GM-CSF and IL-17 in peripheral CD4^+^ T cell subpopulations during multiple sclerosis relapses and remission. Impact of corticosteroid therapy

**DOI:** 10.1371/journal.pone.0173780

**Published:** 2017-03-16

**Authors:** Nathalie Muls, Zakia Nasr, Hong Anh Dang, Christian Sindic, Vincent van Pesch

**Affiliations:** 1 Unité de Neurochimie, Institute of Neuroscience, Avenue Mounier, 53 (BP B1.53.03), Université catholique de Louvain, Brussels, Belgium; 2 Cliniques Universitaires Saint-Luc, Neurology Department, avenue Hippocrate, 10. Brussels Belgium; Jackson Laboratory, UNITED STATES

## Abstract

Multiple sclerosis (MS) is thought to be a Th17-mediated dysimmune disease of the central nervous system. However, recent publications have questioned the pathogenicity of IL-17 per se and rather suggest the implication of other Th17-related inflammatory mediators. Therefore, we studied the expression of GM-CSF, IL-22, IL-24, IL-26 and CD39 in peripheral blood mononuclear cells (PBMCs) from MS patients during relapses, remission and following corticosteroid treatment. We performed qPCR to measure mRNA levels from *ex vivo* or *in vitro*-stimulated PBMCs. Cytokine levels were determined by ELISA. We used flow cytometry to assess GM-CSF^+^, IL-22^+^ and CD39^+^ cells in relationship to IL-17^+^ CD4^+^ T cells. Our results showed that IL-22 mRNA and IL-22^+^CD4^+^ lymphocytes are increased in circulating cells of relapsing MS patients compared to remitting patients while GM-CSF was unchanged. We have further shown that 12.9, 39 and 12.4% of Th17 cells from MS patients during relapses expressed IL-22, GM-CSF and CD39 respectively. No changes in these proportions were found in stable MS patients. However, the majority of GM-CSF^+^ or IL-22^+^ T cells did not co-express IL-17. GM-CSF mRNA, but not IL-22 mRNA, was dramatically decreased *ex vivo* by ivMP. Our results contribute to a better characterisation of Th17, Th22 and ThGM-CSF cells in the setting of MS and according to disease activity.

## Introduction

Multiple sclerosis (MS) is a chronic multifocal inflammatory disease of the central nervous system (CNS). MS lesions are characterised by immune cell infiltrates, demyelination, axonal degeneration and astrogliosis. Early in disease pathogenesis, repair mechanisms compensate ongoing CNS damage. Over time, these mechanisms become insufficient, resulting in the accumulation of permanent disability.

Th17 cells have been implicated in MS pathogenesis. These cells are characterised by the production of proinflammatory cytokines such as IL-17A, IL-17F, IL-21, IL-22 and GM-CSF. Several lines of evidence highlight the involvement of Th17 cells in experimental autoimmune encephalomyelitis (EAE) and MS. In EAE for instance, IL-17 deficient mice show delayed onset and decreased disease severity and disease cannot be induced in IL-23p19 or IL-23R knock-out mice [1, 2], suggesting a strong implication of the IL-23/IL-17 pathway in CNS autoimmunity. In MS, higher levels of IL-17 mRNA and protein have been detected in PBMCs and cerebrospinal fluid (CSF) cells [3–5]. However, the mechanisms by which Th17 cells are pathogenic are still not fully understood.

Some findings question however the primary role of IL-17 in the pathogenicity of Th17 cells. Notably, IL-17-deficient mice can still develop EAE, although with a milder course, and IL-17 neutralization only attenuates the disease [1]. Conversely, as GM-CSF-deficient mice are completely resistant to EAE induction, this cytokine could be crucial to disease pathogenesis [6]. In humans, GM-CSF can promote monocytic migration across the blood-brain barrier and a pro-inflammatory phenotype in CCR2^+^ monocytes [7, 8].

Th17 cells have also been shown to produce IL-22. Together with IL-24 and IL-26, IL-22 belongs to the IL-10 cytokine family. In the murine setting, Th17 cells seem to be the major IL-22 producers [9]. In humans, a cell linage distinct from Th1, Th2 and Th17 has been described and named Th22 [10]. Although IL-22 knock-out mice remain fully susceptible to EAE, the role of IL-22 needs to be clarified since a single-nucleotide polymorphism located near *IL22RA2* has been established as an MS risk factor [11].

Th17 cells with immunosuppressive functions have been identified in several human diseases [12–14]. These cells express the ectonucleotidase CD39 which degrades extracellular ATP to AMP. As ATP stimulation of immune cells induces largely proinflammatory responses such as activation of the inflammasome and subsequent IL-1β maturation, its degradation by CD39 contributes to decrease the inflammatory microenvironment [15]. Furthermore, AMP is in turn rapidly degraded to adenosine by CD73. Adenosine has general inhibitory effects on lymphocytic migration into inflamed areas through A2A receptor signalling [16].

Glucocorticoids (GC), particularly intravenous methylprednisolone (ivMP) are used since the early 1950s and still represent the agent of choice in the treatment of acute MS relapses. Their therapeutic effects are related to multiple immunosuppressive mechanisms, acting in various ways to decrease cytokine production, immune cell extravasation and to induce apoptosis. In the setting of MS relapses, GC could have a dual role, by inhibiting proinflammatory processes [17], but also by increasing the production of anti-inflammatory molecules such as CD39, IL-10 and TGF-β [18–20].

Th17 cells and their related cytokines such as GM-CSF and IL-22 have been repeatedly linked to MS and EAE. However, the cytokine expression profile of Th17 cells in the setting of MS and according to disease activity is not fully described. In this study, we aimed to characterize Th17-associated cytokines during MS relapses in comparison to the remitting phase of the disease and to HC (healthy controls). Furthermore, we evaluated the impact of ivMP treatment on these immunological markers.

## Material and methods

### Subjects

The study was approved by the local ethics committee (Comité d’Ethique hospitalo-facultaire, Cliniques Universitaires Saint-Luc, Université Catholique de Louvain) and written informed consents were obtained from all patients. A total of 80 patients with MS, according to revised McDonald’s Criteria, were enrolled in the study [21]. Blood samples were used for the different experiments. Patients were between 18 and 63 years old. Blood samples were collected from 35 healthy controls (HC). Clinical and demographic features are summarized in Table 1.

**Table 1:** Main demographic features of MS patients and healthy control groups.

**A**	**n**	**Mean age**	**SD**	**% Female**	**Disease duration (years)**	**MSSS**
**Relapsing MS**	57	35.0	10.3	77.4	5,35 (0,01–37)	2,23 (0,1–7.8)
**Stable MS**	23	45.9	9.0	77.3	12.67 (2–27)	2.93 (0–7.59)
**HC**	35	34.9	10.3	71.4	NA	NA
**B**		**Mean (min-max)**				
**pre-relapse EDSS**^b^		0 (0–2.5)				
**Relapse EDSS**^b^		2 (1–4)				
**Relapse duration (days)**		13.26 (1–60)				
**new MRI lesions** ^a^^**,**^ ^b^		2 (0–4)				
**Gd-enhanced lesions**^b^		1 (0–10)				

(A) Mean age, standard deviation of age (years), % of female, disease duration and MS severity score (MSSS) for each patient subgroups. (B) Clinical characteristics of relapsing MS patients.

a) Not applicable for Clinically isolated syndrome patients, Not done for 9 Relapsing-Remitting MS patients

b) Median of the group

23 patients had a stable disease (Stable MS) without any acute clinical event or disability progression at least 6 months before sampling. Four stable patients were treated with glatiramer acetate. They were included in the study, since GA did not affect cytokine expression at the mRNA level (van Pesch et al., unpublished data). Blood samples were collected from a total of 57 relapsing patients (Relapsing MS) who presented with a mono- or multifocal neurological deficit, compatible with MS, lasting more than 24 hours, which was not associated with fever or infection, before administration of ivMP (1g/day for 3 or 5 days). Among these patients, none was previously treated with a disease-modifying drug. 27 samples were collected from relapsing MS patients before the last administration of ivMP on day 5 (ivMP MS). Stable patients were older than relapsing patients. Several years of follow-up are required to determine that a patient with clinically definite MS is stable and to allow eventually suspension of immunomodulatory treatment. This delay accounts for the age difference observed between the patients recruited in the relapsing group and those included in the stable cohort.

### Blood samples collection and PBMC culture

For serum studies, blood samples were collected in S-Monovette tubes (Sarstedt), centrifuged at 3600 rpm for 6 min, aliquoted and stored at -80°C for later analysis.

Peripheral blood mononuclear cells (PBMCs) were prepared by Ficoll-Paque PLUS (GE Healthcare) density gradient centrifugation. Cells were stored in liquid nitrogen until further use in sterile freezing solution containing 60% of RPMI1640 medium (Gibco), 30% of sterile heat-inactivated human serum and 10% of dimethyl sulfoxyde. Vials were thawed in 37°C water bath and washed with RPMI1640 medium with 10% fetal calf serum (Gibco). PBMCs were cultured in X-VIVO^TM^10 medium (Lonza) with IL-2 (5U/ml). For *in vitro* MP analysis, PBMCs from healthy individuals were cultured in presence or absence of 1μM of MP during 24h. Cells were then stimulated during 2h with PHA.

### cDNA synthesis and qPCR

mRNA were extracted from PBMCs either *ex vivo*, without any *in vitro* culture, or after 4h of stimulation with PMA (50ng/ml) and ionomycin (500ng/ml). RNA was isolated using the RNeasy mini kit (Qiagen) according to the manufacturer's protocol. Reverse transcription and qPCR assays were performed as previously described [17]. The relative amount of transcripts was determined by normalizing to Abelson gene (ABL) using the comparative Ct method (2^-ΔΔCt^). ABL mRNA levels were not affected by ivMP treatment. Results are expressed relative to the mean of the healthy control patients set at 1. Scatter dot plots show relative mRNA expression levels in PBMCs. Primer sequences are detailed in S1 Table.

### ELISA

Cytokine concentrations were determined in duplicate using sandwich enzyme immunoassays for quantitative measurement in the patient serum and/or the supernatant following manufacturer instructions. GM-CSF was measured in serum samples and culture supernatants using a sandwich DuoSet ELISA (RandD Systems). Culture supernatants were collected after 4h of stimulation with PMA (50ng/ml) and ionomycin (500ng/ml). Detection range started at 15.6pg/ml. IL-22 concentrations were measured using the Quantikine IL-22 Immunoassay (R&D Systems) in serum and culture supernatants collected after overnight stimulation with a polyclonal stimulation reagent CytoStim (Miltenyi Biotec). The minimal detection level was 2.7pg/mL. IL-26 concentration in the serum was determined using an ELISA kit for human IL-26 (Gentaur). The minimal detectable level of IL-26 was 5.5 pg/mL.

### FACS

PBMCs were stimulated with PMA (50ng/ml) and ionomycin (500ng/ml) in the presence of GolgiStop (BD Biosciences) for 4h. Cells were fixed and permeabilized according to the manufacturer’s instructions (BD Cytofix/Cytoperm; BD Biosciences,). Anti-human monoclonal antibodies used for surface staining were: anti-human CD3, CD4 and CD39 (BioLegend). The following antibodies were used to perform intracellular cytokine staining: GM-CSF, IL-17 (BioLegend) and IL-22 (eBioscience). Both unstained and unstimulated cells were used as controls. Compensations have been set up using OneComp eBeads (eBioscience). Gating strategy is detailed in S1 Fig. Data were acquired in duplicate on a LSR Fortessa instrument (BD Biosciences) and analysed using the FlowJo software (Tree Star Inc.).

### Statistical analysis

All statistical analyses were performed with GraphPad Prism 5 software. To test for differences before and after five days of ivMP treatment as well as for in vitro experiments with MP, non-parametric Wilcoxon’s signed rank tests were performed. Relapsing and stable MS patients and HC were compared using non-parametric one-way ANOVA (Kruskal-Wallis test) followed by a post-test analysis (Dunn’s multiple comparison tests). P-values ≤ 0.05 were considered statistically significant.

## Results

### *Ex vivo* GM-CSF mRNA and GM-CSF-producing cells are similar in relapsing or stable MS and in HC

**Fig 1:**
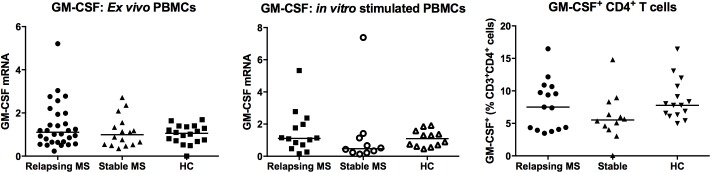
GM-CSF mRNA and GM-CSF-producing cells in PBMCs from relapsing and stable MS patients and from healthy controls (HC). Quantitative-PCR to measure GM-CSF mRNA expression (A) *ex vivo* (Relapsing MS: n = 30, Stable MS: n = 16, HC: n = 18) and (B) after 4h of stimulation by PMA/ionomycin (Relapsing MS: n = 14, Stable MS: n = 10, HC: n = 12). (C) PBMCs were stained for GM-CSF, CD3 and CD4 after 4h stimulation by PMA/ionomycin in the presence of a protein transport inhibitor and analysed by flow cytometry (Relapsing MS: n = 15, Stable MS: n = 12, HC: n = 15). Scatter dot plot illustrates the percentage of GM-CSF^+^CD4^+^ cells. The horizontal lines of scatter plots represent the median value in all subgroups.

Data obtained from EAE studies suggest a role for GM-CSF in autoimmune neuroinflammation. Therefore, we wanted to analyse the expression of this cytokine in MS patients, according to clinical disease activity. GM-CSF was not quantifiable by ELISA in the vast majority of the serum samples analysed (16/18 relapsing MS patients, 17/17 stable patients and 18/19 HC, data not shown). *Ex vivo* GM-CSF transcript levels in PBMCs were comparable between MS patients and HC (Fig 1A, Table 2).

**Table 2:** GM-CSF expression analysis in PBMCs of MS patients and HC.

GM-CSF	Relapsing MS		Stable MS		Healthy controls		ANOVA p-value
	Median	Range	Median	Range	Median	Range	
***Ex vivo* PBMCs mRNA levels**	1.11	0.23–5.21	0.99	0.35–2.72	1	0–1.69	0.6
***In vitro* stimulated PBMC mRNA levels**	1.11	0.16–5.33	0.47	0.13–7.38	1	0.46–1.92	0.13
**GM-CSF**^**+**^ **cells (% CD4**^**+**^ **T cells)**	7.51	3.48–16.47	5.52	0.08–14.8	7.77	4.98–16.43	0.11

GM-CSF mRNA was quantified by qPCR either *ex vivo* or following 4h *in vitro* stimulation by PMA/ionomycin. Results are expressed relative to the mean of the healthy control patients set at 1. GM-CSF^+^CD4^+^ T cells were quantified by FACS upon stimulation in the presence of a protein transport inhibitor. Relapsing and stable MS patients and HC were compared using non-parametric one-way ANOVA (Kruskal-Wallis test).

In order to evaluate GM-CSF expression in response to T-cell activation, cellular mRNA levels and GM-CSF concentration in culture supernatants were determined. Although the level of GM-CSF tends to decrease in stable MS patients, the difference was not statistically significant (Fig 1B). Of note, we observed that the mRNA and protein levels were correlated (Spearman’s r = 0.495, p = 0.0085; data not shown) but were not correlated with age (Spearman’s r = 0.05, p = 0.78), despite the age difference between the stable and remitting patient cohorts.

We next aimed to quantify the frequency of GM-CSF-producing T helper cells. To do so, GM-CSF^+^CD4^+^ T cells were analysed by Fluorescence-activated cell sorting (FACS) following in vitro stimulation by PMA (Phorbol-12-Myristate-13-Acetate) and ionomycin. In agreement with the previous results, the proportion of GM-CSF^+^CD4^+^ T cells was comparable in relapsing and remitting MS as well as in control subjects (Fig 1C, Table 2).

### *Ex vivo* IL-22 mRNA and IL-22-producing T cells are increased in relapsing compared to remitting MS

**Fig 2:**
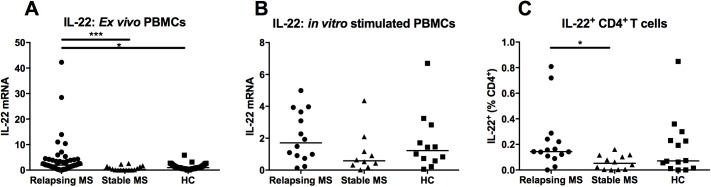
IL-22 mRNA and IL-22-producing cells in PBMCs from relapsing and stable MS patients and from healthy controls (HC). Quantitative-PCR to measure IL-22 mRNA expression (A) *ex vivo* (Relapsing MS: n = 56, Stable MS: n = 16, HC: n = 37) and (B) after 4h of stimulation by PMA/ionomycin (Relapsing MS: n = 14, Stable MS: n = 10, HC: n = 12). (C) PBMCs were stained for IL-22, CD3 and CD4 after 4h stimulation by PMA/ionomycin in the presence of a protein transport inhibitor and analysed by flow cytometry (Relapsing MS: n = 15, Stable MS: n = 12, HC: n = 14). Scatter dot plot shows the percentage of IL-22^+^CD4^+^ T cells. The horizontal lines of scatter plots represent the median value in all subgroups. * and *** indicate respectively p-values ≤ 0.05 and ≤ 0.001.

The identification of a single-nucleotide polymorphism downstream of the *IL22RA2* gene as an MS risk loci as well as the implication of IL-22 in various immune-mediated diseases led us to study this cytokine according to MS disease activity. We measured the *ex vivo* expression of the IL-22 transcript in total PBMCs from relapsing and remitting MS patients as well as from HC (Fig 2A, Table 3). IL-22 mRNA was significantly increased in relapsing patients in comparison to stable patients (p = 0.0002) and to HC (p = 0.0025). The median mRNA level in patients experiencing a MS relapse was 7.1 and 2.13 times higher than in stable individuals and in control subjects respectively. There was no correlation between the mRNA expression level of IL-22 and clinical characteristics such as age, duration of relapse, localization of relapse, EDSS at the time of relapse, Multiple Sclerosis Severity Score, lesion load on MRI assessed by the Barkhof criteria, presence or absence of spinal cord lesion on MRI, presence or absence of gadolinium enhancement on MRI, presence or absence of oligoclonal bands in the CSF, clinical status (Clinically isolated syndrome, patients fulfilling the 2010 McDonald criteria for MS or patients with clinically definite MS) and disease duration. IL-22 was undetectable by Enzyme-linked immunosorbent assay (ELISA) (<15.6pg/ml) both in the serum and in the CSF of MS patients (data not shown).

**Table 3:** IL-22 expression analysis in PBMCs of MS patients and HC.

IL-22	Relapsing MS		Stable MS		Healthy controls		ANOVA p-value	Dunn's Multiple Comparison Test		
	Median	Range	Median	Range	Median	Range		Relapsing MS vs HC	Relapsing MS vs Stable MS	HC vs Stable MS
***ex vivo* PBMC mRNA levels**	2	0–42.27	0.29	0–2.59	0.96	0–5.84	< 0.0001	*	***	ns
***in vitro* stimulated PBMC mRNA levels**	1.71	0.13–4.99	0.59	0.02–4.36	1.2	0.06–6.7	0.15	NA	NA	NA
**IL-22**^**+**^ **cells (% CD4**^**+**^ **T cells)**	0.14	0–0.81	0.05	0–0.16	0.25	0.85	0.03	ns	*	ns

IL-22 mRNA was quantified by qPCR either *ex vivo* or *in vitro* after 4h stimulation by PMA/ionomycin. Results are expressed relative to the mean of the healthy control patients set at 1. IL-22^+^CD4^+^ T cells were quantified by FACS upon PMA/ionomycin stimulation in the presence of a protein transport inhibitor. Relapsing and stable MS patients and HC were compared using non-parametric one-way ANOVA (Kruskal-Wallis test). In case of a significant ANOVA, post-test analysis was performed (Dunn’s multiple comparison tests). In the figures

* and *** indicate p-values of ≤0.05 and ≤0.001 respectively. NA not applicable, ns non significant.

Upon polyclonal stimulation, PBMCs from MS patients and HC produced IL-22 mRNA at comparable levels although there was a tendency for decrease in IL-22 messenger in stable MS patients (Fig 2B). In order to investigate IL-22 secretion by PBMCs from relapsing patients, IL-22 concentration was measured in culture supernatants. PBMCs from patients and controls secreted IL-22 in the same range (90–1300 pg/ml) following activation (data not shown). We then evaluated the proportion of IL-22-producing T helper within the circulating cells. Using flow cytometry, we found that the percentage of IL-22^+^CD4^+^ T cells was significantly increased during MS relapses in comparison to the stable phase of the disease (Fig 2C, Table 3).

### *Ex vivo* IL-24 and IL-26 mRNA are comparable in relapsing or stable MS, and in HC

IL-24 and IL-26 are members of the IL-10 family of cytokines just as IL-22. IL-22 and IL-24 have been shown to play redundant functions because they share the same cytokine receptor subunit IL-22R1. In addition, human Th17 have been shown to produce IL-26 after differentiation from naïve CD4^+^ T cells. Therefore, *ex vivo* IL-24 and IL-26 mRNA were analysed but no difference in PBMCs transcript levels was found between MS patients and HC (S2A and S2B Fig). Similarly, following PBMC stimulation, mRNA levels were similar between relapsing, stable patients and HC (S2C and S2D Fig).

The hypothesis that IL-26 levels could vary in the serum of MS patients has recently emerged. Therefore, we measured IL-26 in serum samples from patients and controls. Although no difference between groups was detected, IL-26 concentrations ranged from 0.75 to 70.73 pg/ml (S2E Fig).

### GM-CSF, IL-22 and CD39 expression by Th17 cells during MS relapses

**Fig 3:**
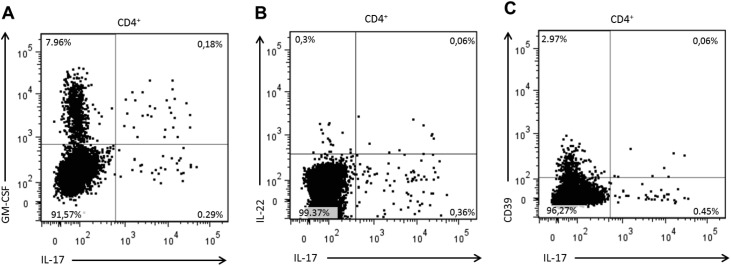
FACS analysis of GM-CSF, IL-22 and CD39 coexpression with IL-17 in CD4^+^ T cells. PBMCs were fixed and stained after 4h stimulation by PMA/ionomycin in the presence of a protein transport inhibitor. CD4^+^ cells were gated among the CD3^+^ population. Representative dot plots of (A) GM-CSF, (B) IL-22 and (C) CD39 costaining with IL-17. Numbers in each quadrant represent the average percentage of positive CD4^+^ T cells in relapsing MS patients.

We aimed to further characterise the expression of GM-CSF, IL-22 and CD39 by Th17 lymphocytes. The proportion of Th17 lymphocytes expressing GM-CSF, IL-22 and CD39 were comparable between relapsing and stable MS patients (S3 Fig and S2 Table). In humans, GM-CSF producing-cells could represent a Th17-independent subtype of T cells. To explore whether Th17 cells express GM-CSF, we assessed the co-expression of those cytokines. On average, 8.14% of CD4^+^ T cells expressed GM-CSF (Fig 3A, Table 4). 7.96% of the CD4^+^ T cells produced GM-CSF alone while 0.18% of CD4^+^ T cells were GM-CSF^+^IL-17^+^, forming a distinct cell subpopulation. Thus, 38.99% of the IL-17^+^CD4^+^ T cells were positive for GM-CSF.

**Table 4:** Characterisation of GM-CSF, IL-22 and CD39 expression by Th17 lymphocytes during MS relapses.

**(A)**	**% of CD4**^**+**^ **T cells**
**IL-17**^**+**^	**0.47**
**GM-CSF**^**+**^	**8.14**
**GM-CSF**^**+**^**IL-17**^**-**^	**7.96**
**GM-CSF**^**+**^**IL-17**^**+**^	**0.18**
**IL-22**^**+**^	**0.36**
**IL-22**^**+**^**IL-17**^**-**^	**0.3**
**IL-22**^**+**^**IL-17**^**+**^	**0.06**
**CD39**^**+**^	**3.03**
**CD39**^**+**^**IL-17**^**-**^	**2.97**
**CD39**^**+**^**IL-17**^**+**^	**0.06**
**(B)**	**% of IL-17**^**+**^**CD4**^**+**^ **T cells**
**GM-CSF**^**+**^ **IL-17**^**+**^	**38.99**
**IL-22**^**+**^ **IL-17**^**+**^	**12.94**
**IL-22**^**+**^ **GM-CSF**^**+**^ **IL-17**^**+**^	**9.27**
**CD39**^**+**^ **IL-17**^**+**^	**12.36**

PBMCs were stimulated during 4h with PMA/ionomycin in the presence of a protein transport inhibitor. (A) The mean percentages of CD4^+^ T cells expressing GM-CSF, IL-22 and CD39 alone or in combination with IL-17 are presented. (B) Mean percentage of IL-17^+^CD4^+^ T cells expressing GM-CSF, IL-22 and CD39.

IL-22 could be part of the Th17 cytokine signature or alternatively be produced by an independent subset of T cells (Th22). Furthermore, under certain conditions, the proinflammatory properties of IL-22 could depend on its coproduction with IL-17. Therefore, we intended to characterize IL-17 and IL-22 coproduction. PBMCs were isolated from MS patients and stimulated with PMA and ionomycin. On average, 0.36% of CD4^+^ cells were positive for IL-22. 0.3% of CD4^+^ T cells expressed IL-22 without IL-17 while only 0.06% coexpressed the two cytokines (Fig 3B, Table 4). Based on these figures, double positive cells represented 12.94% of IL-17^+^ CD4^+^ T cells. On average, 9.27% of Th17 lymphocytes simultaneously expressed IL-22 and GM-CSF.

In contrast to pathogenic Th17, Th17 with suppressive activity have been described. These suppressive Th17 express high levels of the ectonucleotidase CD39. Therefore, we intended to investigate whether IL-17^+^CD4^+^ T cells expressed CD39 during MS relapses. As illustrated in Fig 3C, 3.03% of the CD4^+^ T cells expressed CD39 on their cellular membrane following stimulation. CD39^+^ cells represented 12.36% of Th17 lymphocytes (Table 4).

### Impact of ivMP on GM-CSF and IL-22

**Fig 4:**
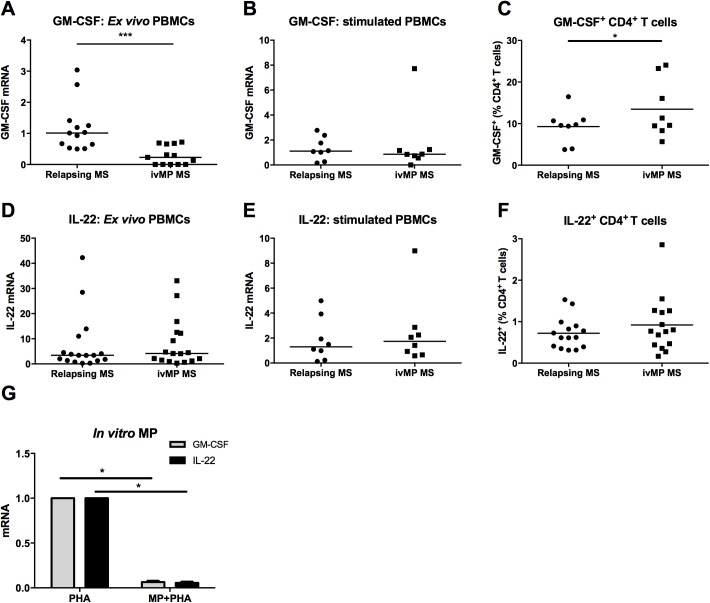
GM-CSF and IL-22 before and after intravenous methylprednisolone (ivMP) treatment in paired samples of relapsing MS patients. Quantitative-PCR to measure GM-CSF and IL-22 mRNA expression *ex vivo* (A, n = 13 and D, n = 17) and (B, n = 8 and E, n = 8) after 4h stimulation by PMA/ionomycin. Results are expressed relative to the mean of the relapsing patients set at 1. PBMCs were stained for GM-CSF (C, n = 8) or IL-22 (F, n = 15), CD3 and CD4 after 4h stimulation by PMA/ionomycin in the presence of a protein transport inhibitor and analysed by flow cytometry. The horizontal lines of scatter plots represent the median value in all subgroups. (G) PBMCs of HC (n = 5) were treated with methylprednisolone (MP) for 24h and stimulated 2h with PHA. Error bars represent the standard error of the mean. * and *** indicate respectively p-values ≤ 0.05 and ≤ 0.001.

IvMP is a potent anti-inflammatory agent that exerts multiple effects on the immune system. Here, we intended to further investigate the impact of ivMP on IL-22 and GM-CSF. First, we measured the *ex vivo* mRNA expression levels in total PBMCs from relapsing MS patients before and after 5 days of ivMP treatment (Fig 4, S3 Table). We then quantified mRNA expression and the frequency of IL-22 and GM-CSF-producing CD4^+^ T cells in response to polyclonal stimulation by FACS.

GM-CSF mRNA was decreased by 80% 5 days after ivMP treatment in circulating cells of MS patients (Fig 4A). However, following *in vitro* polyclonal stimulation, GM-CSF mRNA was expressed at similar levels independently of previous exposure to ivMP (Fig 4B). Paradoxically, the treatment significantly increased the frequency of GM-CSF-producing CD4^+^ T cells by approximatively 10% (Fig 4C).

Surprisingly, the mRNA expression of IL-22 was not influenced by 5 days of ivMP therapy both *ex vivo* and following *in vitro* stimulation (Fig 4D and 4E). Similarly, the proportion of IL-22^+^CD4^+^ T cells did not change after *in vivo* exposure to MP (Fig 4F). These results suggest that i*n vitro* activated PBMCs respond to the stimulation independently of previous *in vivo* exposure to MP.

To analyse further the effects of MP on IL-22 and GM-CSF, PBMCs were cultured in the presence of 1μM of MP during 24h. Cells were then stimulated during 2h. These experiments clearly indicated that *in vitro* treatment of PBMCs with MP decreased the expression of both IL-22 and GM-CSF mRNA (Fig 4G). Similar results were obtained for IL-26 (data not shown).

## Discussion

Since their discovery, Th17 cells and their signature cytokine IL-17 have been largely incriminated in the development of EAE and MS [22, 23]. However, the pathogenicity of IL-17-producing T helper cells cannot be attributed to this single cytokine. For instance, IL-17-deficient mice are not protected against EAE induction but show delayed disease onset and decreased EAE severity [1]. On the contrary, GM-CSF -/- mice are completely resistant to EAE induction [6]. Therefore, GM-CSF has emerged as a possible mediator of Th17 pathogenicity, notably through the recruitment of myeloid cells and the induction of a pro-inflammatory phenotype in CCR2^+^ monocytes [8]. Consequently, GM-CSF neutralizing antibodies have been developed and are evaluated as a novel therapeutic approach for MS [24]. In this study, we evaluated the expression level of GM-CSF from relapsing and stable MS patients. GM-CSF mRNA and protein levels are comparable between MS patients and healthy controls. Considering these results, GM-CSF alone cannot serve as a biomarker of disease activity. Some authors have suggested that GM-CSF levels are increased in the CSF of MS patients in comparison to neuromyelitis optica and healthy subjects [25]. However, in our experiments, we could not detect measurable levels of this cytokine in CSF samples.

Similarly to GM-CSF, IL-22 is also considered as a Th17-related cytokine. Although the understanding of the cellular source and molecular regulation of IL-22 has significantly progressed in recent years, its role in MS remains to be clarified. In 2011, Almolda and colleagues investigated the kinetics of IL-22 levels in acute EAE model. They showed that the level of IL-22 was high during the induction phase of EAE while it was markedly decreased during recovery [26]. However, IL-22 knockout mice immunized with MOG peptides developed EAE similar to wild type controls [27]. This result suggests that IL-22 is not directly involved in disease pathogenesis in the murine model of MS. In humans, a study conducted on a small cohort of five patients suggested an increased number of IL-22-producing cells in active MS [5]. Further investigations revealed an elevated number of Th22 cells, particularly in relapsing MS [28]. In addition, a single-nucleotide polymorphism downstream of the *IL22RA2* gene was associated with an increased MS risk [11]. Here, we show an increase in *ex vivo* IL-22 mRNA expression from PBMCs of relapsing MS patients. However, in agreement with Rolla *et al*., we did not find any correlation between IL-22 expression and any disease characteristics [28]. We could also observe a decrease of IL-22 producing CD4^+^ T cells in stable compared to relapsing patients. IL-22 is elevated in the serum of patients with psoriasis, Crohn‘s disease and Guillain-Barré syndrome [29–31]. In our hands, it was undetectable both in the serum and CSF samples of MS patients.

IL-22 is a member of the IL-10 family of cytokines, also including IL-24 and IL-26. IL-22 and IL-24 share the cytokine receptor subunit IL-22R1 and these cytokines have been shown to play redundant functions [32]. To our knowledge, IL-24 has never been studied in the context of MS but this cytokine is likely involved in other chronic inflammatory-mediated diseases. For instance, studies on psoriasis and inflammatory bowel disease suggest a role of IL-24 in disease pathogenesis [33, 34]. Similarly to GM-CSF, IL-26 can be secreted by human Th17 cells. Of note, intriguingly, the IL-26 gene is absent in mice. Only a few studies have analysed IL-26 in MS but it has been suggested that the level of this cytokine is higher in the serum of MS patients [35]. However, we did not find any statistically significant difference in neither IL-24 nor IL-26 expression at the mRNA level between MS patients and HC. Therefore, the observed increase in IL-22 mRNA during MS relapses is unique among members of this cytokine family.

As previously mentioned, IL-22 and GM-CSF can be secreted by Th17 cells and influence their pathogenicity. Here, we aimed to further characterize Th17 cells in the context of MS. Therefore, we isolated PBMCs from MS patients and analysed the coexpression of IL-17 with IL-22, GM-CSF and CD39 by CD4^+^ T cells. We report that nearly 40% of the IL-17^+^CD4^+^ T cells also produce GM-CSF in our experimental conditions. In mice, GM-CSF is usually described as a Th17-related cytokine [36]. However, it has recently been suggested that naïve murine CD4^+^ T cells could be differentiated in a GM-CSF^+^ cellular subset distinct from Th1 and Th17 cells [37]. In humans, experimental data have suggested that GM-CSF rather associates with the Th1 profile [38]. According to our results, most GM-CSF-positive CD4^+^ T cells do not express IL-17. However, in 2011, Hirota and co-workers showed that murine Th17 cells could shut down IL-17 expression and progressively deviate to IL-17^-^ T cells that produce other inflammatory cytokines [39]. Thus, GM-CSF^+^ IL-17^-^ T cells could still potentially originate from Th17 cells.

IL-22 has a dual role in inflammation, mediating proinflammatory properties in psoriasis but protective functions in hepatitis and inflammatory bowel disease [40–42]. These different functions for IL-22 are likely dependent on the inflammatory context. The presence of IL-17 could direct the pathogenic *versus* protective role of IL-22 [43, 44]. IL-22 could synergize with IL-17 to promote inflammation but divergent conclusions have been reported [43, 45]. Therefore, we investigated IL-17 and IL-22 coproduction in relapsing MS patients. In our FACS study, immunostaining of IL-22 in IL-17-producing CD4^+^ T cells was low. Indeed, less than one out of a thousand CD4^+^ T cells coproduce these cytokines. Consequently, IL-22 seems to be mainly produced by other cell subsets (Th22) rather than Th17. In line with this result, in an acute EAE model using Lewis rats immunized with MBP, Th17 cells did not produce IL-22 [26].

We have previously demonstrated an increase in the proportion of CD39^+^ Tregs in relapsing patients [20]. An upregulation of CD39 mRNA level was also observed at the peak of EAE [46]. In addition, Th17 cells expressing CD39 could play immunoregulatory roles by converting extracellular ATP to AMP. To our knowledge, these CD39^+^IL-17^+^ cells have not been studied in MS patients. Therefore, we attempted to quantify this cell population in circulating PBMCs of patients. We found that CD39^+^ cells represented only a minor fraction (11.8%) of Th17 lymphocytes.

Finally, we studied the impact of corticosteroid treatment administered during MS relapses on GM-CSF and IL-22. We have shown previously that inflammatory cytokines such as IL-23, IL-6 and IFN-γ were down-regulated following ivMP [17]. In contrast, IL-10 and CD39 were increased after the treatment [17, 20]. Our results demonstrate that IL-22 mRNA as well as IL-22-producing CD4^+^ cells are likely not influenced by *in vivo* exposure to MP. This is, to our knowledge, the first report regarding the effect of ivMP on IL-22 in MS patients. Disparate observations have been made concerning the effect of GC treatment on IL-22 in other human diseases. These discrepancies are likely caused by variability in dosing, exposure duration, route of administration or type of GC used [47]. In addition, several mechanisms of GC resistance have been described, among which the expression of the P-glycoprotein/multi-drug resistance type 1 in Th17 cells [48, 49].

Regarding the effect of GC on GM-CSF, we have shown that this treatment decreased *ex vivo* GM-CSF mRNA levels in PBMCs but not in response to cellular activation. Surprisingly, the proportion of GM-CSF^+^CD4^+^ T cells noticeably increased following ivMP. A higher resistance of GM-CSF^+^ CD4^+^ T cells to GC-induced apoptosis could potentially explain this observation. These conflicting results led us to analyse the influence of MP on cell cultures. The addition of MP in the culture medium clearly shut down both GM-CSF and IL-22 mRNA levels. Therefore, based on the results of *in vitro* exposure to MP, the direct effect of this drug on the transcription of these two cytokines is likely to be inhibitory. However, our results suggests that glucocorticoid resistance mechanisms are induced following *in vivo* exposure of IL-22-expressing CD4^+^ T cells to ivMP, that are not present when cells are primed *in vitro* with MP.

In conclusion, the diagnosis of MS and more specifically of disease activity is still hampered by the lack of specific immunological biomarkers. Identification of such markers would be a useful tool to guide therapeutic decisions in MS. In addition, these biomarkers could serve in disease monitoring and assessment of therapeutic efficacy. Our results indicate that the IL-22 transcript is increased in relapsing MS patients. It could also be produced by other cell types such as innate lymphoid cells, as recently suggested by Gross et al [50]. Currently, we do not know the physiological meaning of this increase. Further analyses are conceivable but appear challenging. In fact, as IL-22R is not expressed on immune cells, the downstream targets of IL-22 cannot be investigated from human blood samples. Potential targets could rather be located within the CNS such as astrocytes [51].

In summary, although IL-22 and GM-CSF are classically described as Th17-associated cytokines, we have shown that they are mainly not co-expressed with IL-17 in CD4^+^ T cells from MS patients. The proportion of GM-CSF^+^ CD4^+^ T cells is comparable between relapsing and stable MS patients, whereas Th22 cells and IL-22 mRNA are increased during MS relapses. Regarding the effects of corticosteroids, discrepant *in vivo* versus *in vitro* effects were found for both IL-22- and GM-CSF-expressing cells, hinting towards the presence of GC resistance mechanisms in these cells. These results warrant further investigation into the functionality and targets of the complex T helper cell response in the setting of MS, to allow the development of more specific and targeted therapeutic strategies.

## Supporting information

S1 Table Primers sequences used for qPCR amplification. (DOCX)

S2 Table Characterisation of GM-CSF, IL-22 and CD39 expression by Th17 lymphocytes in relapsing and stable MS patients and HC. PBMCs were stimulated during 4h with PMA/ionomycin in the presence of a protein transport inhibitor. (A) The average percentages of CD4^+^ T cells expressing IL-17, GM-CSF, IL-22 and CD39 are presented. (B) The average percentages of GM-CSF-, IL-22- and CD39-expressing cells within the IL-17^+^CD4^+^ T cell population are indicated.
(DOCX)

S3 Table Summary of the *in vivo* and *in vitro* effects of methylprednisolone on GM-CSF and IL-22 compared with untreated samples. (DOCX)

S1 Fig Gating strategy used to analyse CD4^+^ T cells by FACS. (A) Lymphoid cells were selected according to their SSC/FSC profile. (B) Cellular doublets were excluded of the analysis using FSC-Width plotted against FSC-Height. (C) CD3^+^ cells were selected within single cells using histogram representation. (D) CD4^+^ cells were selected within CD3^+^ population using histogram.
(TIFF)

S2 Fig IL-24 and IL-26 in MS patients. Quantitative-PCR to measure: (A) *ex vivo* IL-24 mRNA levels in relapsing MS (n = 35) and HC (n = 18). (B) ex vivo IL-26 mRNA levels in relapsing MS (n = 15) and HC (n = 10). (C) IL-24 (Relapsing MS: n = 14, Stable MS: n = 10, HC: n = 13) and (D) IL-26 (Relapsing MS: n = 14, Stable MS: n = 10, HC: n = 13) mRNA expressions after 4h of PMA/ionomycin stimulation. (E) IL-26 concentration was quantified by ELISA in serum (Relapsing MS: n = 10, Stable MS: n = 10, HC: n = 10).
(TIFF)

S3 Fig GM-CSF, IL-22 and CD39-expressing Th17 cells from relapsing and stable MS patients and from healthy controls (HC). PBMCs were stained for IL-17, GM-CSF, IL-22, CD39, CD3 and CD4 after 4h stimulation by PMA/ionomycin in the presence of a protein transport inhibitor and analysed by flow cytometry. Scatter dot plots illustrate the percentage of GM-CSF^+,^ IL-22^+^ and CD39^+^ cells within the IL-17^+^CD4^+^ T cell population. The horizontal lines of scatter plots represent the median value in all subgroups.
(TIFF)
