# Supplementary material for: IL-22, GM-CSF and IL-17 in peripheral CD4+ T cell subpopulations during multiple sclerosis relapses and remission. Impact of corticosteroid therapy
Source: PLoS One. 2017 Mar 16;12(3):e0173780. doi: 10.1371/journal.pone.0173780 (PMC5354390; doi:10.1371/journal.pone.0173780)
Supplement: S1 Table — (DOCX) [file pone.0173780.s001.docx]

S1 Table.

| Target | Forward primer (5'-3') | Reverse Primer (5'-3') |
| --- | --- | --- |
| ABL | AAAACCTTCTCGCTGGACCC | TTTGGGCTTCACACCATTCC |
| CD39 | GTGGAGTTCAAAATAGACATCGTG | CAGCACCCACACCGCCTTCTCCCGCTT |
| IL-22 | AGGCTCAGCAACAGGCTAAG | TTTGCTCTGGTCAAATGCAG |
| IL-24 | CAGGGTGTGGACAAGGTAACA | CTCAGGATAACATCACGAGTGC |
| IL-26 | GGAAGACGTTTTTGGTCAACTGC | CTCTCTAGCTGATGAAGCACAGG |
| GM-CSF | GGGAGCATGTGAATGCCATC | GGCTCCTGGAGGTCAAACAT |
