# Supplementary material for: IL-22, GM-CSF and IL-17 in peripheral CD4+ T cell subpopulations during multiple sclerosis relapses and remission. Impact of corticosteroid therapy
Source: PLoS One. 2017 Mar 16;12(3):e0173780. doi: 10.1371/journal.pone.0173780 (PMC5354390; doi:10.1371/journal.pone.0173780)
Supplement: S2 Table — PBMCs were stimulated during 4h with PMA/ionomycin in the presence of a protein transport inhibitor. (A) The average percentages of CD4+ T cells expressing IL-17, GM-CSF, IL-22 and CD39 are presented. (B) The average percentages of GM-CSF-, IL-22- and CD39-expressing cells within the IL-17+CD4+ T cell population are indicated. (DOCX) [file pone.0173780.s002.docx]

S2 Table.

| **A. % of CD4^+^ T cells** | **Relapsing MS** | **Stable MS** | **HC** |
| --- | --- | --- | --- |
| IL-17^+^ | 0.47 | 0.16 | 0.36 |
| GM-CSF^+^ | 8.14 | 6.03 | 8.63 |
| IL-22^+^ | 0.36 | 0.06 | 0.17 |
| CD39^+^ | 3.03 | 6.18 | 2.29 |
| **B. % of IL-17^+^CD4^+^ T cells** | |  |  |
| GM-CSF^+^ IL-17^+^ | 38.99 | 40.11 | 44.21 |
| IL-22^+^ IL-17^+^ | 12.94 | 13.66 | 9.80 |
| IL-22^+^ GM-CSF^+^ IL-17^+^ | 9.27 | 11.96 | 8.54 |
| CD39^+^ IL-17^+^ | 12.36 | 25.07 | 13.88 |
