# Supplementary material for: IL-22, GM-CSF and IL-17 in peripheral CD4+ T cell subpopulations during multiple sclerosis relapses and remission. Impact of corticosteroid therapy
Source: PLoS One. 2017 Mar 16;12(3):e0173780. doi: 10.1371/journal.pone.0173780 (PMC5354390; doi:10.1371/journal.pone.0173780)
Supplement: S3 Table — (DOCX) [file pone.0173780.s003.docx]

S3 Table.

|  | **GM-CSF** | **IL-22** |
| --- | --- | --- |
| **mRNA from *ex vivo* PBMCs** | ↓ | = |
| **mRNA from polyclonally Stimulated PBMCs**  **(following *in vivo* exposure to ivMP)** | = | = |
| **% of CD4^+^ T cells**  **(following *in vivo* exposure to ivMP)** | ↑ | = |
| **mRNA from stimulated PBMCs**  **(following *in vitro* exposure to ivMP)** | ↓↓↓ | ↓↓↓ |
