# Supplementary figures and images for: IL-22, GM-CSF and IL-17 in peripheral CD4+ T cell subpopulations during multiple sclerosis relapses and remission. Impact of corticosteroid therapy
Source: PLoS One. 2017 Mar 16;12(3):e0173780. doi: 10.1371/journal.pone.0173780 (PMC5354390; doi:10.1371/journal.pone.0173780)

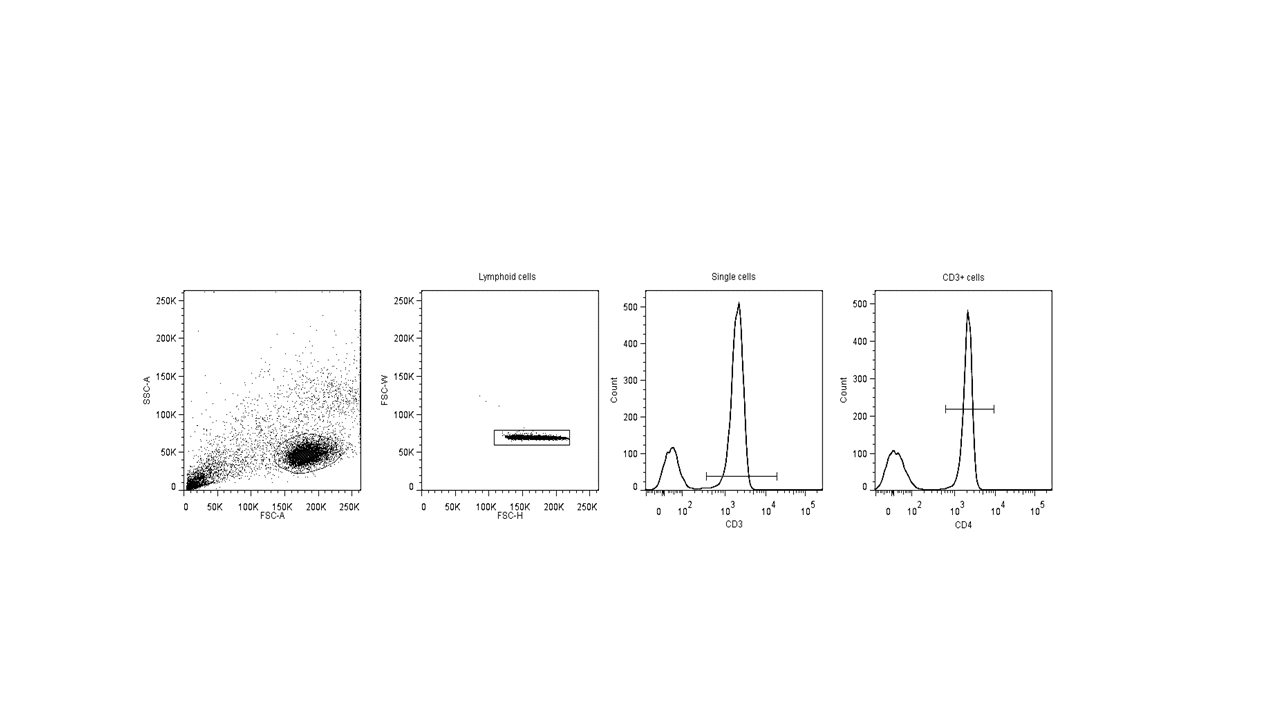

Supplement: S1 Fig — (A) Lymphoid cells were selected according to their SSC/FSC profile. (B) Cellular doublets were excluded of the analysis using FSC-Width plotted against FSC-Height. (C) CD3+ cells were selected within single cells using histogram representation. (D) CD4+ cells were selected within CD3+ population using histogram. (TIFF) [file pone.0173780.s004.tiff]

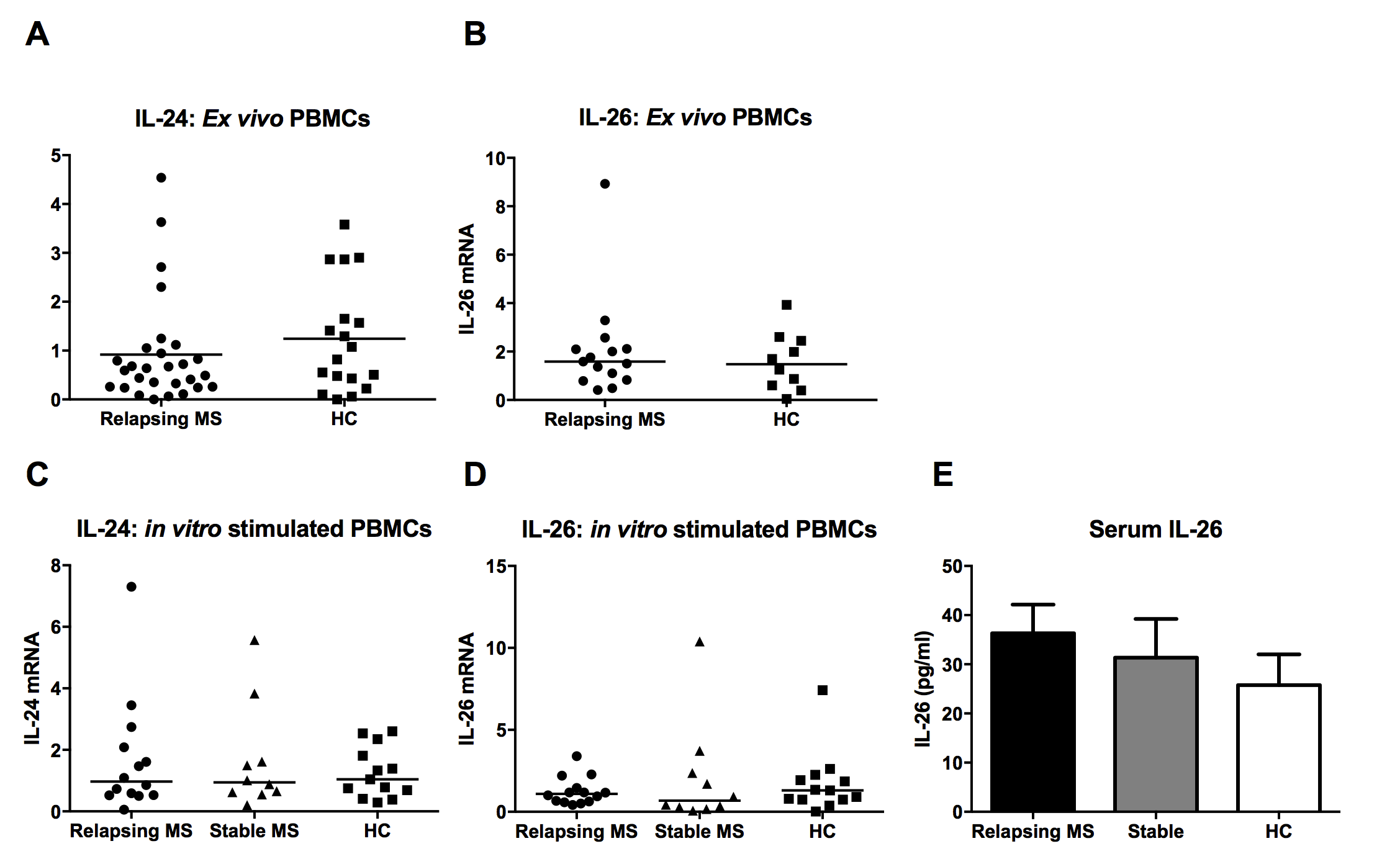

Supplement: S2 Fig — Quantitative-PCR to measure: (A) ex vivo IL-24 mRNA levels in relapsing MS (n = 35) and HC (n = 18). (B) ex vivo IL-26 mRNA levels in relapsing MS (n = 15) and HC (n = 10). (C) IL-24 (Relapsing MS: n = 14, Stable MS: n = 10, HC: n = 13) and (D) IL-26 (Relapsing MS: n = 14, Stable MS: n = 10, HC: n = 13) mRNA expressions after 4h of PMA/ionomycin stimulation. (E) IL-26 concentration was quantified by ELISA in serum (Relapsing MS: n = 10, Stable MS: n = 10, HC: n = 10). (TIFF) [file pone.0173780.s005.tiff]

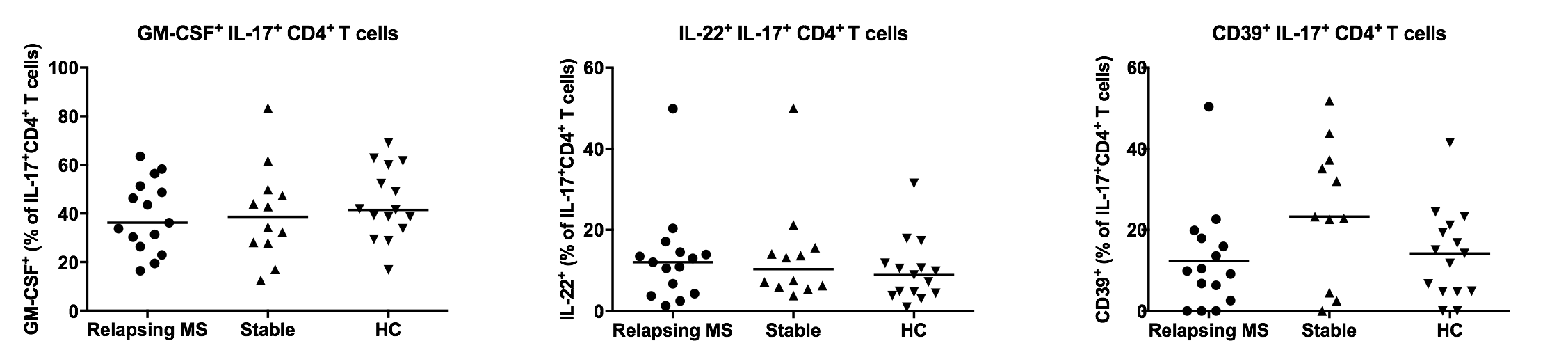

Supplement: S3 Fig — PBMCs were stained for IL-17, GM-CSF, IL-22, CD39, CD3 and CD4 after 4h stimulation by PMA/ionomycin in the presence of a protein transport inhibitor and analysed by flow cytometry. Scatter dot plots illustrate the percentage of GM-CSF+, IL-22+ and CD39+ cells within the IL-17+CD4+ T cell population. The horizontal lines of scatter plots represent the median value in all subgroups. (TIFF) [file pone.0173780.s006.tiff]
